# Replication of Integrative Data Analysis for Adipose Tissue Dysfunction, Low-Grade Inflammation, Postprandial Responses and OMICs Signatures in Symptom-Free Adults

**DOI:** 10.3390/biology10121342

**Published:** 2021-12-16

**Authors:** Esther C. Gallegos-Cabriales, Ernesto Rodriguez-Ayala, Hugo A. Laviada-Molina, Edna J. Nava-Gonzalez, Rocío A. Salinas-Osornio, Lorena Orozco, Irene Leal-Berumen, Juan Carlos Castillo-Pineda, Laura Gonzalez-Lopez, Claudia Escudero-Lourdes, Judith Cornejo-Barrera, Fabiola Escalante-Araiza, Eira E. Huerta-Avila, Fatima A. Buenfil-Rello, Vanessa-Giselle Peschard, Eliud Silva, Rosa A. Veloz-Garza, Angelica Martinez-Hernandez, Francisco M. Barajas-Olmos, Fernanda Molina-Segui, Lucia Gonzalez-Ramirez, Ruy D. Arjona-Villicaña, Victor M. Hernandez-Escalante, Janeth F. Gaytan-Saucedo, Zoila Vaquera, Monica Acebo-Martinez, Areli Murillo-Ramirez, Sara P. Diaz-Tena, Benigno Figueroa-Nuñez, Melesio E. Valencia-Rendon, Rafael Garzon-Zamora, Juan Manuel Viveros-Paredes, Salvador B. Valdovinos-Chavez, Anthony G Comuzzie, Karin Haack, Ashley A. Thorsell, Xianlin Han, Shelley A. Cole, Raul A. Bastarrachea

**Affiliations:** 1 Facultad de Enfermería, Universidad Autónoma de Nuevo León (UANL), Monterrey 64460, Mexico; esther.gallegosc@gmail.com (E.C.G.-C.); rosyvelozgarza@hotmail.com (R.A.V.-G.); svmonterrey@gmail.com (S.B.V.-C.); 2 Centro de Investigación en Ciencias de la Salud (CICSA), Facultad de Ciencias de la Salud, Universidad Anáhuac Norte, Lomas Anahuac 52786, Mexico; Ernesto.rodriguez@anahuac.mx (E.R.-A.); fescara@gmail.com (F.E.-A.); giselle.peschard@gmail.com (V.-G.P.); jose.silva@anahuac.mx (E.S.); 3 Escuela de Ciencias de la Salud, Universidad Marista de Mérida, Mérida 97300, Mexico; hlaviada@marista.edu.mx (H.A.L.-M.); nutfernandamolina@gmail.com (F.M.-S.); lucia.gonzalez.ramirez@emory.edu (L.G.-R.); ruyarjona@gmail.com (R.D.A.-V.); hescalan@correo.uady.mx (V.M.H.-E.); 4 Facultad de Salud Pública y Nutrición (FASPyN), UANL, Monterrey 64460, Mexico; edna.navag@uanl.mx; 5 Departamento de Nutrición, Universidad del Valle de Atemajac (UNIVA), Zapopan 45050, Mexico; rocio.salinas@univa.mx (R.A.S.-O.); laura.gonzalez@univa.mx (L.G.-L.); tantarrea@gmail.com (M.E.V.-R.); garzonzam120@hotmail.com (R.G.-Z.); juan.viveros@univa.mx (J.M.V.-P.); 6 Laboratorio de Inmunogenómica y Enfermedades Metabólicas, Instituto Nacional de Medicina Genómica, SS, Ciudad de México 14610, Mexico; lorozco@inmegen.gob.mx (L.O.); lneeha920609@gmail.com (E.E.H.-A.); amartinez@inmegen.gob.mx (A.M.-H.); fbarajas@inmegen.gob.mx (F.M.B.-O.); 7 Facultad de Medicina y Ciencias Biomédicas, Universidad Autónoma de Chihuahua, Chihuahua 31125, Mexico; ileal@uach.mx; 8 Departamento de Nutrición Humana, Universidad Latina de América, Morelia 58170, Mexico; castillomorelia@gmail.com (J.C.C.-P.); areli.murillo@gmail.com (A.M.-R.); sara.diaztena@gmail.com (S.P.D.-T.); 9 Facultad de Ciencias Químicas, Universidad Autónoma de San Luis Potosí, San Luis Potosi 78240, Mexico; clauescu@yahoo.com (C.E.-L.); monica.acebo@uaslp.mx (M.A.-M.); 10 Departamento de Enseñanza, Postgrado e Investigación, Hospital Infantil de Tamaulipas, Ciudad Victoria 87150, Mexico; jcornejob@hotmail.com; 11 Population Health Program, Southwest National Primate Research Center (SNPRC), Texas Biomedical Research Institute, San Antonio, TX 78227-0549, USA; fatimabuenfilnutricion@gmail.com (F.A.B.-R.); janethgaytan@gmail.com (J.F.G.-S.); zvaquera@txbiomed.org (Z.V.); khaack@txbiomed.org (K.H.); scole@txbiomed.org (S.A.C.); 12 Clínica de Enfermedades Crónicas y Procedimientos Especiales (CECYPE), Morelia 58249, Mexico; benigno.figueroa@cecype.com; 13 The Obesity Society, Silver Spring, MD 20910, USA; tcomuzzie@obesity.org; 14 Sansum Diabetes Research Institute, Santa Barbara, CA 93105, USA; athorsell@sansum.org; 15 Department of Medicine, Sam and Ann Barshop Institute for Longevity and Aging Studies, University of Texas Health San Antonio, San Antonio, TX 78229, USA; hanx@uthscsa.edu

**Keywords:** metabolically healthy/unhealthy phenotype, adipose tissue dysfunction, postprandial inflammatory response, low-grade chronic subclinical inflammation, fat and muscle tissue biopsies, OMICs molecular signatures, symptom-free volunteers

## Abstract

**Simple Summary:**

There is an alarming increase of cardiovascular disease (CVD) and type 2 diabetes (T2D) in Mexican nationals and Mexican Americans. Studying adipose tissue (AT) dysfunction and early biomarkers of cardiovascular and immunometabolic risk in Mexican nationals may have a strong impact on future public health policies for US-born Mexican Americans and other populations of Mexican origin in the US. The goal of this study is to evaluate the early transition towards healthy/unhealthy adipose tissue expansion to identify AT dysfunction through systemic, molecular and OMICS measures in the fasting and fed states in symptom-free volunteers with no history of age-related chronic diseases in support of precision medicine and discovery.

**Abstract:**

We previously reported preliminary characterization of adipose tissue (AT) dysfunction through the adiponectin/leptin ratio (ALR) and fasting/postprandial (F/P) gene expression in subcutaneous (SQ) adipose tissue (AT) biopsies obtained from participants in the GEMM study, a precision medicine research project. Here we present integrative data replication of previous findings from an increased number of GEMM symptom-free (SF) adults (N = 124) to improve characterization of early biomarkers for cardiovascular (CV)/immunometabolic risk in SF adults with AT dysfunction. We achieved this goal by taking advantage of the rich set of GEMM F/P 5 h time course data and three tissue samples collected at the same time and frequency on each adult participant (F/P blood, biopsies of SQAT and skeletal muscle (SKM)). We classified them with the presence/absence of AT dysfunction: low (<1) or high (>1) ALR. We also examined the presence of metabolically healthy (MH)/unhealthy (MUH) individuals through low-grade chronic subclinical inflammation (high sensitivity C-reactive protein (hsCRP)), whole body insulin sensitivity (Matsuda Index) and Metabolic Syndrome criteria in people with/without AT dysfunction. Molecular data directly measured from three tissues in a subset of participants allowed fine-scale multi-OMIC profiling of individual postprandial responses (RNA-seq in SKM and SQAT, miRNA from plasma exosomes and shotgun lipidomics in blood). Dynamic postprandial immunometabolic molecular endophenotypes were obtained to move towards a personalized, patient-defined medicine. This study offers an example of integrative translational research, which applies bench-to-bedside research to clinical medicine. Our F/P study design has the potential to characterize CV/immunometabolic early risk detection in support of precision medicine and discovery in SF individuals.

## 1. Introduction

Certain individuals can be considered metabolically healthy despite their long-standing and high degree of body fat accumulation. This effect can be thought of as healthy AT expansion. On the other hand, unhealthy AT expansion is a major contributor to the systemic metabolic disturbances that are characteristic of obesity and type 2 diabetes mellitus (T2DM) [1]. The loss of expansion capacity can occur in patients with normal weight, explaining the existence of MUH lean subjects [2,3].

Maintaining a body mass index (BMI) in the ideal range of 20.0–25.0 kg/m^2^ may effectively reduce the risk of early death from cardiovascular and immunometabolic disease [4]. In addition, subjects exhibiting MH obesity (MHO) are not at increased risk of cardiovascular events compared to MH people in the normal weight range. It appears that, independent of BMI, AT dysfunction [5] triggers early molecular events that ultimately lead both to insulin resistance (IR) and also to low-grade chronic subclinical inflammation (LGCSI) and subsequent morbidity and mortality. The recently developed ALR correlates with IR better than adiponectin or leptin alone [6]. This emerging biomarker decreases with increasing number of cardiometabolic risk factors, reflecting the functionality of AT, and negatively correlates with markers of LGCSI [7]. The ALR has been suggested as a significant marker of AT dysfunction and inflammation [8,9].

Cardiovascular Disease (CVD) and T2DM are serious public health problems among Mexican nationals [10] and Mexican Americans [11]. Identifying MH/MUH parameters occurring simultaneously with AT dysfunction markers in Mexican nationals will enhance the understanding of early underlying biology of T2DM and CVD [12,13]. The knowledge gained will have a strong impact on future public health policies to decrease the burden of T2DM and CVD in Hispanics, Mexican Americans and populations of Mexican origin in the US.

The GEMM family study (Genética de las Enfermedades Metabólicas en México, Genetics of Metabolic Diseases in Mexico) is a bi-national, multi-center collaborative study of cardiovascular risk phenotypes of immunometabolic origin (CVRIMO) related to the risk of T2DM and CVD. The GEMM study is a longitudinal, family-based, precision medicine research project. In addition, known relatedness provides one level of informed adjustment for admixture, an important consideration given the diverse ancestry of modern Mexicans and many other cohorts. GEMM has acquired data directly from three tissues that are highly relevant to immunometabolism: AT, SKM and blood (immune system). Our measurements in blood and tissues were taken over a time course to allow fine scale multi-OMIC profiling of individual postprandial responses. Unlike studies utilizing tissue repositories of poorly-characterized origin, our study obtained tissues from a well-characterized cohort of symptom-free adults of common ancestry [14,15].

Precision and personalized medicine, linked to the identification of early risk and prevention instead of curative pathological symptoms, is rapidly taking place in the immunometabolic and CVD field. This paper aims to identify early biomarkers of immunometabolic dysregulation in persons without clinical symptoms, which may define a future MUH phenotype, and at characterizing the AT dysfunction phenotype at a postprandial systemic and molecular level in SF individuals from the GEMM study. Our working hypothesis is that by comparing the metabolically healthy vs. unhealthy phenotype in our SF participants and their ALR levels, we will be able to screen for presence or absence of systemic immunometabolic dysfunction. We will also be able to identify in them, within the normal variation, significant differences in circulating levels of proinflammatory cytokines and the presence of impaired postprandial peaks from hormones related to the insulin–glucose axis. This paper increases and replicates previous published data from 80 individuals, with a focus on subcutaneous AT profiling [15].

## 2. Methods

Recruitment of study participants: As of February 2020, GEMM had performed the meal challenge, tissue biopsies and sample collection for 225 participants [14,15]. Unfortunately, the COVID-19 pandemic forced us to temporarily stop recruitment. However, we were able to bring samples from 124 study participants to the US in December 2019 and have obtained data from these recruited participants (81 females and 43 males). Our cohort is comprised of SF volunteers that were carefully screened to rule out presence of any chronic or acute inflammatory disease, as described in detail elsewhere [14,15,16].

Mixed Meal Challenge: GEMM participants were given an innovative balanced mixed-meal challenge [17]. We provided a mixed meal in the form of Ensure^®^ Plus liquid shake from Abbott Laboratories (macronutrient composition: 65% carbohydrate, 15% protein and 20% fat) dosed at 30% of each participant’s resting energy expenditure in Kcal/day. This dosage mimics the first morning meal after an overnight fast and allows each participant to receive his/her own ideal calorie amount as dictated by his/her own resting metabolic rate and expenditure. Our goal was to compare the metabolic response to a fixed macronutrient composition among each participant [18]. We chose a liquid shake instead of a solid meal for easier means to administer the mixed meal challenge.

Phenotyping Methodology: We recruited study participants according to established guidelines and strategies [19]. We chose to initially classify our SF participants with the presence/absence of adipose tissue dysfunction into two groups according to their ALR cut-off point > 1 or <1 [7,9]. The different experimental groups were established through adiponectin and leptin circulating measures to determine the adiponectin/leptin ratio (ALR) trait and to characterize adipose tissue dysfunction. The rationale for this approach is that a low ALR as a marker of AT dysfunction is characterized by a lower secretion of adiponectin in relation to leptin levels, triggering unhealthy adipose tissue hypoxia, proinflammatory macrophage polarization traits, altered adipokine profile and insulin resistance (IR). Bioimpedance was measured by Tanita BC—418 Body Composition Analyzer, and body composition by dual energy X-ray absorptiometry. A wide range of clinical biochemistries, hormones, cytokines and endophenotypes were measured for F/P metabolic assessments through analyte categories, including β-cell and the insulin–glucose axis, inflammation endophenotypes and lipid–lipoprotein metabolism. These biochemical phenotypes were analyzed on a Luminex 100IS platform and an Immulite 1000 which runs enzyme-linked immunosorbent assay (ELISA) and radioimmunoassay (RIA) analyses.

Shotgun Lipidomics: Lipidomics analysis serves as a powerful tool for understanding the biochemical and cellular mechanisms underlying lipid-related immunometabolic disease processes by quantifying the changes of individual lipid classes, subclasses and molecular species [20]. Plasma samples were transported to Dr. Xianlin Han’s laboratory at the University of Texas Health Sciences Center San Antonio [21]. Lipids were extracted with chloroform/methanol in the presence of a cocktail of internal standards [22] and analyzed by multi-dimensional mass spectrometry-based shotgun lipidomics (MDMS-SL), as previously described [14,15,23].

miRNA Sequencing from plasma exosomes: Exosomal miRNA was isolated from 1 mL of plasma using the Plasma/Serum Exosome Purification and RNA Isolation Mini Kit (Norgen, Thorold, ON, Canada). The quality and quantity of miRNA were assessed using the Low Abundance RNA Quantification Kit (Norgen). Conversion of miRNA into a cDNA library was performed using the NEXTflex Illumina Small RNA-Seq Kit v3 (Bio Scientific, Phoenix, AZ, USA). The purification and enrichment of cDNA by PCR was performed to create cDNA libraries. Forty-eight sample libraries were pooled and quantified using the Kapa Library ABI Prism Quantification kit. Library pools were sequenced using a commercial service.

Methodology RNA isolation: Total RNA was isolated from pre- and post-prandial samples for each tissue type. Isolation from adipose and muscle shock frozen tissue used a Direct-zol RNA Miniprep Plus kit w/TRI reagent (Zymo Research, Tustin, CA, USA). Tissue was homogenized in TRI reagent using a Beadbeater (Biospec Products, Bartlesville, OK, USA). Total RNA was isolated from blood samples using the Tempus^TM^ spin RNA isolation kit (ThermoFisher Scientific, Waltham, MA, USA). RNA quantity was determined using a Qubit^TM^ RNA BR assay kit (ThermoFisher Scientific), and RNA quality/integrity was assessed using the RNA screen tape assay (Agilent Technologies, Santa Clara, CA, USA). The GLOBINclear^TM^ kit (ThermoFisher Scientific) was used to isolate globin mRNA from total RNA in blood.

RNASeq of RNA from fat and muscle: mRNA (from total RNA) was converted into a cDNA library using the Kapa mRNA HyperPrep kit for Illumina Platforms and unique dual-indexed adapter kit (Kapa Biosystem, Wilmington, MA, USA). Library quality was checked using D1000 Screen Tapes (Agilent Technologies). Samples were quantified by qPCR using the Kapa Library ABI Prism Quantification kit (Kapa Biosystems) before pooling, and pools were quantified using the same kit after pooling 24 samples to load on one flow cell lane. Library pools were sequenced using a commercial service.

Multidimensional analysis platform development: For this paper, we used FunRich, an open-access, standalone functional enrichment and network analysis tool to perform analysis on background databases that are integrated from heterogeneous genomic and proteomic resources (>1.5 million annotations) [24,25] and the statistical software R version 4.1.1 (R Core Team, 2021, https://www.R-project.org/, accessed on 15 October 2021).

Statistical Analysis: Data are presented as mean ± standard deviation (SD), unless otherwise indicated. Differences between groups are analyzed by two-tailed unpaired Student’s *t*-tests, as appropriate. The calculations were performed using JMP^®^ 14.2.0 (SAS Institute Inc., Cary, NC, USA) and GraphPad Prism 6 (GraphPad Software, Inc., La Jolla, CA, USA). Multivariate linear regression analysis was used to measure the strength of the relationship between our immunometabolic F/P phenotypes with the ALR. For statistical analysis of independent multiple testing, we used a one-way analysis of variance (ANOVA) and Tukey’s multiple comparison test. Our models incorporated the random effect of kinship and included age and sex as covariates. The analysis of the relative gene expression data was conducted by using the delta-delta Ct method to analyze the relative changes in gene expression from our real-time quantitative PCR experiments. This approach relates to the PCR signal of the target transcript in a treatment group to that of another sample such as an untreated control as has been described by Livak and Schmittgen [26]. One-way ANOVA was used to study the association between serum stimulation time and the level of gene expression. Fold increase in mRNA was the dependent variable.

## 3. Results

We chose to initially classify our SF participants with the presence/absence of adipose tissue dysfunction into two groups according to their ALR cut-off point > 1 or <1 [7,9]. We found interesting differences between the high (H)ALR and low (L)ALR groups. Table 1 and Table 2 show the demographic characteristics and immunometabolic parameters for the participants with a (H)ALR or a (L)ALR in our symptom-free (SF) GEMM cohort. Eighty-four individuals had an ALR > 1, and 40 individuals had an ALR < 1. There were significant and striking differences of non-traditional immunometabolic biomarkers [27,28] between individuals with a (H)ALR vs. a (L)ALR. Systemic levels of leptin, weight, waist circumference, % body fat, circulating proinflammatory levels of IL-6, plasminogen activator inhibitor (PAI)-1, hsCRP and fibrinogen and the 5 h postprandial triglyceride curves were significantly increased in the group with AT dysfunction. Our data also showed significantly lower levels of adiponectin. All phenotypes pertaining to the insulin–glucose axis (fasting and 2 h insulin levels, 2 h postprandial glucose levels, fasting glucagon, the Matsuda and the HOMA-IR indexes and curves of 5 h glucose and GLP-1) in the (L)ALR category were impaired when compared with the (H)ALR group (Table 1 and Table 2).

**Figure 1:**
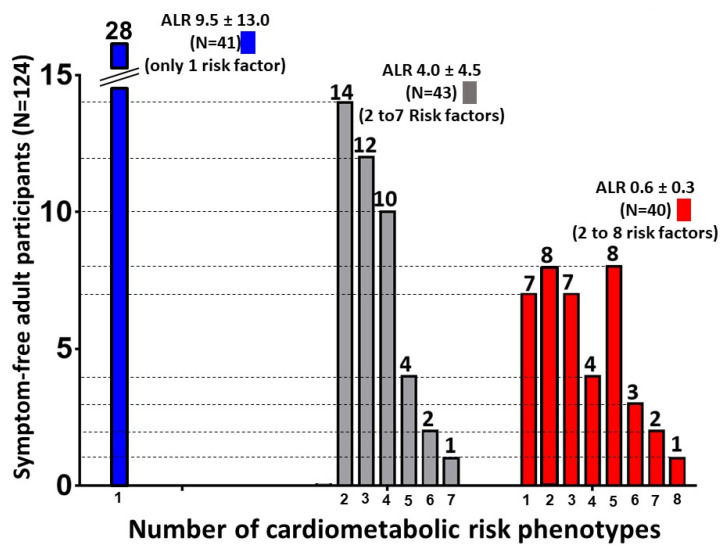
Number of cardiometabolic risk phenotypes among our symptom-free participants (N = 124) stratified by a very high ALR (9.5, N = 41), high ALR (4.0, N = 43) and low ALR (0.6, N = 40).

We estimated the presence/absence of metabolic health (MH and MUH) among the SF GEMM subjects (according to their ALR cut-off point > 1 or <1) using the proposed criteria for defining MH/MUH. This was based on the number of metabolic syndrome parameters present and an assessment of insulin sensitivity (using the Matsuda index score [29]) and LGCSI [30] as determined by hsCRP levels [4,31,32] (Figure 1). We identified three subgroups of symptom-free individuals (Figure 1): (i) Group 1: subjects with a (H)ALR having <2 of the metabolic syndrome risk factors and the absence of IR and LGCSI; (ii) Group 2: subjects with a (H)ALR having ≥2 MUH phenotypes for metabolic risk and the presence of IR and LGCSI; and (iii) Group 3: the same characteristics as Group 2 but with a (L)ALR (Table 3). The proposed criteria to identify MH individuals was based on cutoff values for a healthy cardiometabolic profile [4,29,32,33,34,35]. Reference values in Mexican Americans have been determined with a HOMA-IR > 3.80 as having clear correlates of insulin resistance [36].

**Figure 2:**
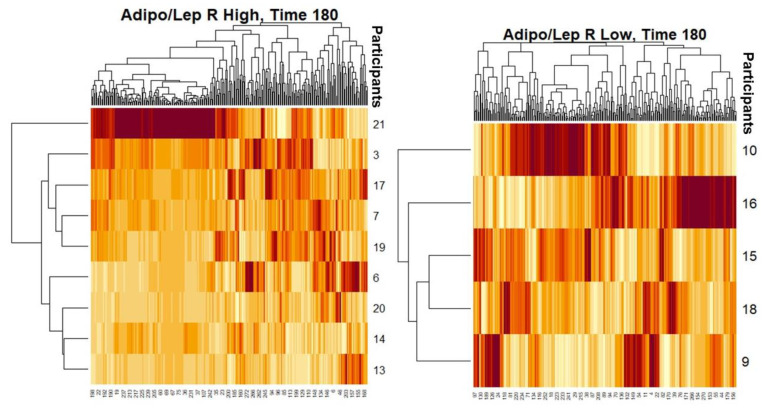
Heat maps with differences in lipidomics signatures for Time 180′ (peak of postprandial response) after a mixed meal among participants with (H) vs. (L)ALR.

**Figure 3:**
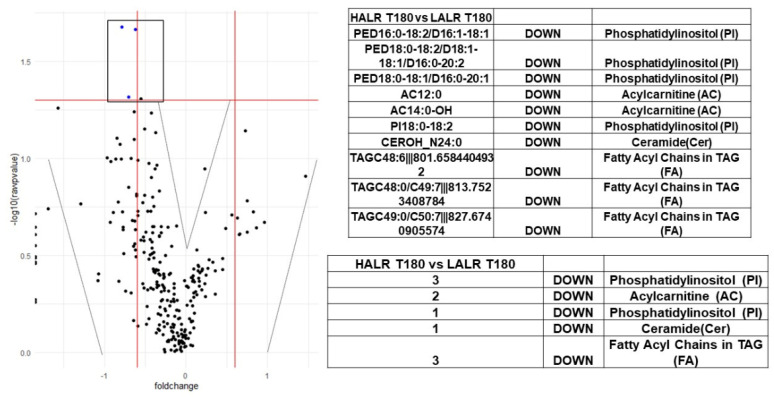
Volcano plot with differences in lipidomic signatures for Time 180′ (peak of postprandial response) after a mixed meal among participants with (H) vs. (L)ALR. Individual lipid classes and species are shown in boxes (right).

**Figure 4:**
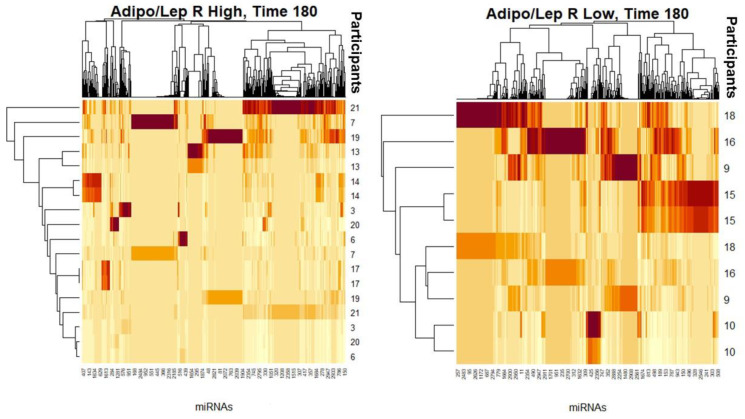
Heat maps with differences in miRNA signatures for Time 180′ (peak of postprandial response) after a mixed meal among participants with (H) vs. (L)ALR.

**Figure 5:**
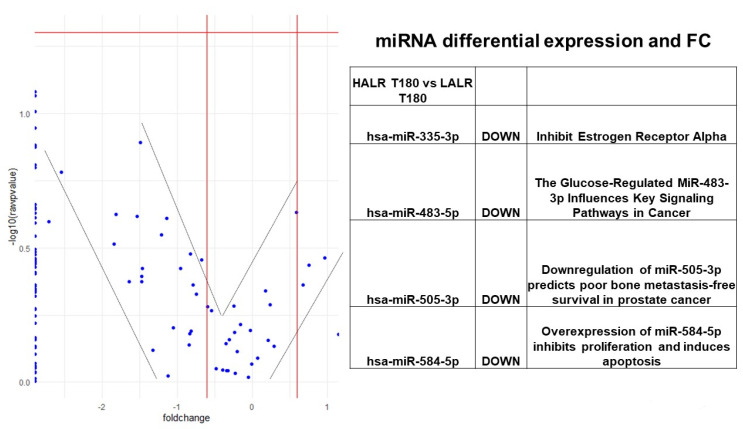
Volcano plot with differences in miRNA signatures for Time 180′ (peak of postprandial response) after a mixed meal among participants with (H) vs. (L)ALR.

We performed shotgun lipidomics and a comprehensive sequencing of exosomal miRNA study using fasting and postprandial plasma samples from a subgroup of 14 females from our SF cohort. Table 4 shows their clinical characteristics. We carefully matched this subgroup by age and fat% from our total SF female cohort (N = 81). We performed whole shotgun lipidomics in fasting and 3 h postprandial plasma. Using these lipidomics results, we classified the 14 females having a high (H; mean 2.2) or low (L; mean 0.5) ALR, and compared the differential signatures of lipid classes, subclasses and molecular species. This strategy allowed identification and quantification of an immense quantity of individual lipid species and classes, including the following: triglycerides, diglycerides, monoglycerides, cholesterol and cholesteryl esters, oxysterols, acylcarnitines, acyl-CoAs, phospholipids (including cardiolipin), lysophospholipids, eicosanoids, 4-hydroxy alkenals and retinoic acid [37]. Shotgun lipidomics and miRNA differential expression profiles are shown in Figure 2, Figure 3, Figure 4 and Figure 5. The implications of these data are explained in the Section 4.

We compared F/P gene expression profiles of two key tissues (SKM and SQAT) between MH and MUH individuals with or without AT dysfunction in 6 SF volunteers (Table 5), chosen from our previous 14 SF adult females, matched by gender and fat% (Table 4).

**Figure 6:**
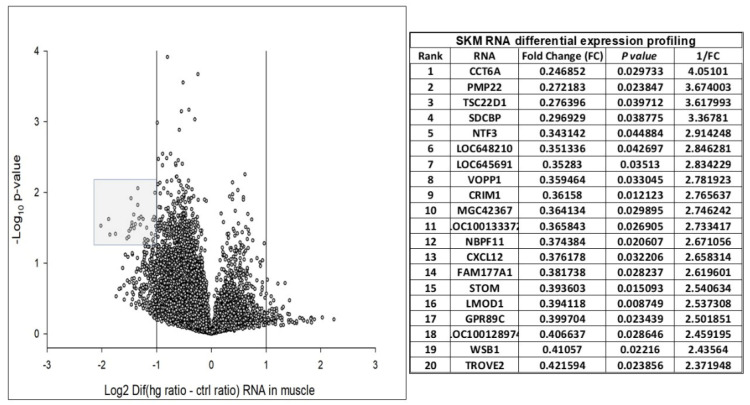
Twenty logarithmic fold change (FC) > 2 and significant *p*-values for skeletal muscle (SKM) differentially expressed genes (DEGs). Volcano plot of FC differences of SKM RNA expression between RG and CG. The vertical lines correspond to FC > 2.

**Figure 7:**
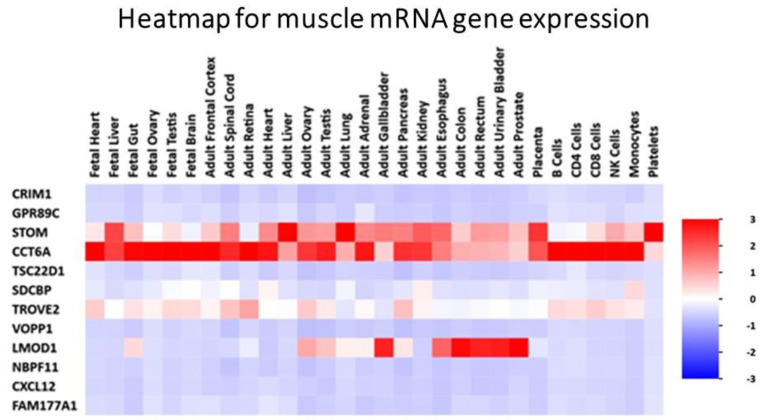
Heat map with differences in gene expression from SKM signatures from the 20 genes identified as shown in Figure 6. Gene CCT6A shows “hot red” values perhaps indicating higher activity in several fetal and adult pathways and, interestingly, from the immune systems through B cells, CDK4, CDK8, monocytes and NK cells.

**Figure 8:**
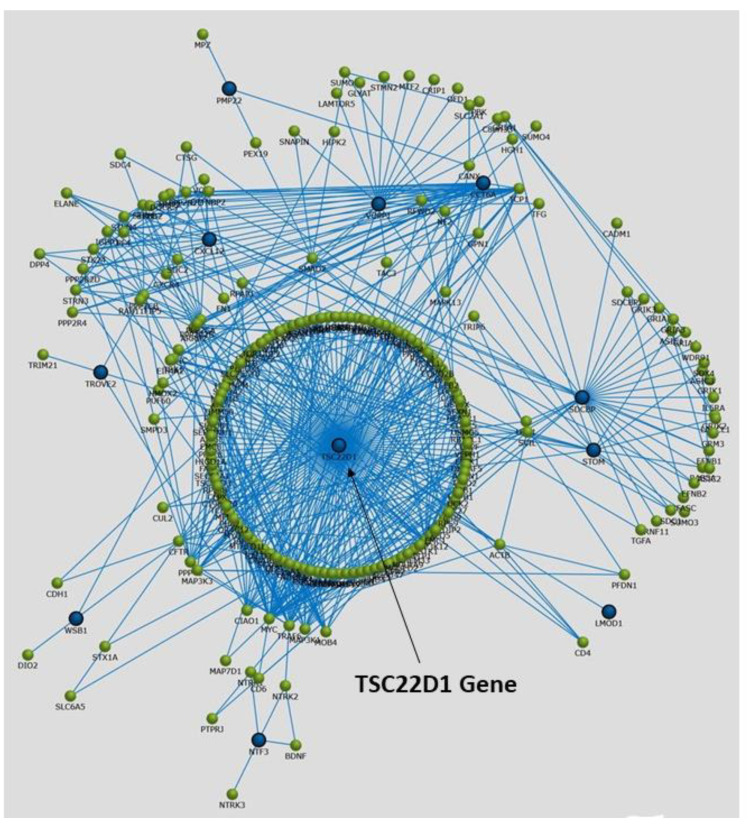
Graphical representation of enrichment analysis and interaction networks obtained through the FunRich database analysis tool for gene annotation pertaining to transcript expression associations in SKM tissue.

**Figure 9:**
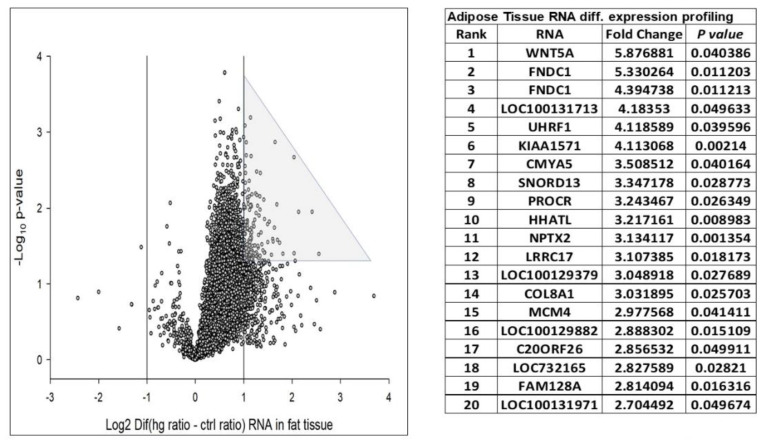
Twenty logarithmic fold change (FC) > 2 and significant *p*-values for adipose tissue (AT) differentially expressed genes (DEGs). Volcano plot of FC differences of AT RNA expression between RG and CG. The vertical lines correspond to FC > 2.

**Figure 10:**
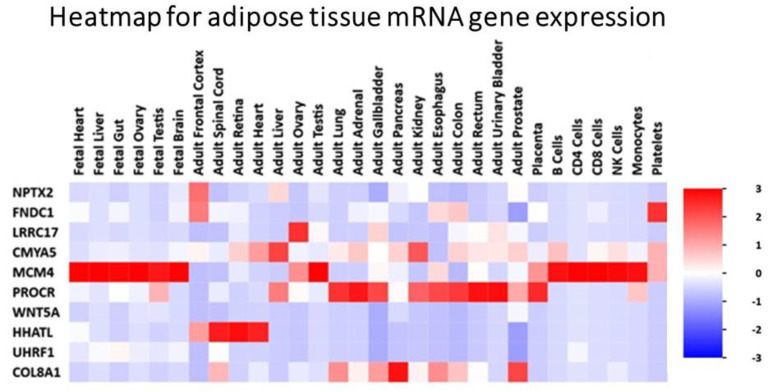
Heat map with differences in gene expression from adipose tissue signatures from the 20 genes identified as shown in Figure 9. Gene MCM4 shows “hot red” values perhaps indicating higher activity in several fetal pathways and, interestingly, from the immune systems through B cells, CDK4, CDK8, monocytes and NK cells.

**Figure 11:**
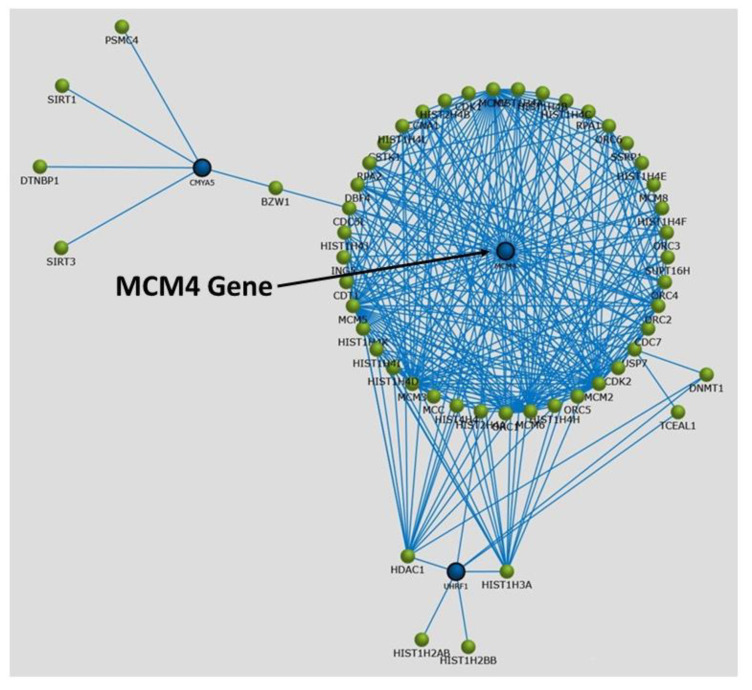
Graphical representation of enrichment analysis and interaction networks obtained through the FunRich database analysis tool for gene annotation pertaining to transcript expression associations in adipose tissue.

We assigned participants with a (L)ALR (mean 0.52) < 1 as the at-risk group (RG) and participants with a (H)ALR (mean 1.98) > 1 as the control (low risk) group (CG) (Table 5). The contrast linear combination of variables between the CG and RG are shown in Table 6. Figure 6 and Figure 9 report differentially expressed genes in SKM (twenty genes) and AT (twenty genes) between the CG vs. RG. By comparing the magnitude of change vs. the statistical significance (*p*-value) we were able to allegedly identify the most biologically significant genes with large fold changes that are statistically significant. Figure 7 and Figure 10 show heat maps with differences in gene expression from SKM and AT signatures from the 20 genes identified for each tissue as shown in Figure 6 and Figure 9. Figure 8 and Figure 11 show the enrichment analysis and interaction networks obtained through the FunRich database analysis tool. The explanation of the associations and gene interactions is written in the Section 4.

## 4. Discussion

In this study, we used a precision medicine screening approach anchored by measurements of AT function and MH/MUH phenotypes among active, symptom-free (SF) adults to identify integrative strategies for detection of early immunometabolic and cardiovascular risk. Our cohort was chosen with a careful exclusion criterion applied for participants with acute illness, activity-limiting unexplained illness, absence of metabolic syndrome criteria, hypertension, dyslipidemia, prevalent diabetes, known cardiovascular or chronic lung disease, cancer or renal failure [16]. We decided to characterize our SF cohort with the presence/absence of AT dysfunction through a novel biomarker, the adiponectin/leptin ratio (ALR) [6,7,9].

As shown in Table 1 and Table 2, we found that one group (N = 84) had a high (H)ALR (6.7 ± 10.0) that translated to a healthy AT expansion and the second group (N = 40) had a low (L)ALR (0.6 ± 0.3) that translated to an unhealthy AT expansion. We found interesting differences between (H) vs. (L)ALR when we characterized AT dysfunction. The anthropometric variables, the β-cell and insulin–glucose axis phenotypes, the adipose tissue hormones, the lipid–lipoprotein and the proinflammatory phenotypes were significantly altered in the (L)ALR group. These findings represent a solid indication that there is an AT metabolic imbalance measured through the plasma ALR in our GEMM SF subjects. However, we should keep in mind that males and females may exhibit differences in adiponectin/leptin ratios and metabolic dysfunction, given that 81 participants were females out of our 124 recruited SF subjects. Our findings also highlight the importance of postprandial immunometabolism through measurements of dynamic phenotypes. After careful screening of AT dysfunction through the ALR, we estimated the presence/absence of MH and MUH risk among our SF GEMM subjects as shown in Figure 1 and Table 3. We were able to combine the MH/MUH phenotype with the AT dysfunction phenptype in our SF cohort. In order to clasify our 124 SF subjects as metabolically healthy, we used well-defined criteria: absence of abdominal obesity based on waist circumference, absence of metabolic syndrome components, normal lipid values, normal fasting glucose concentrations, normal C-reactive protein concentrations and absence of IR as evaluated by the Matsuda index (Table 3) [4,32,38,39].

Most studies regarding metabolic health have focused their reports on individuals with obesity. They have designated that metabolically healthy obesity (MHO) is applied to those who meet the BMI cutoff point of ≥30 kg/m^2^, but do not have other major cardiovascular risk factors and who are not at higher cardiovascular risk than nonobese individuals [40]. One major weakness with respect to this phenotype is that perhaps it solely represents a subgroup of obese subjects with just a lower quantity of metabolic risk than expected for their degree of BMI [41]. Moreover, there are subsets of individuals who are considered “normal/healthy” weight, categorized by a low BMI, but with an increased metabolic/cardiovascular risk. This subset, characterized by elevated insulin levels, elevated triglyceride levels and insulin resistance are much harder to characterize than the MHO group and are predisposed to subsequent development of T2DM and coronary artery disease [42,43,44]. Despite these controversies, we decided to estimate the presence/absence of metabolic health (MH/MUH) among the SF GEMM subjects according to their ALR cut-off points characterizing AT dysfunction. The analysis of the phenotypes associated with MH/MUH are combined for males and females due to their similarity.

Three subgroups of SF individuals were identified: one group with only one metabolic risk phenotype and the absence of IR and LGCSI with a high (H) mean ALR of 9.5 ± 13.0, and a second and third group with altered fasting glucose levels (>100 mg/dL), low HDL-C values, high triglyceride levels, visceral obesity and isulin resistance with a mean ALR of 4.0 ± 4.5 and 0.6 ± 0.3, respectively (Table 3, Figure 1). Twenty-eight individuals fulfilled the criteria of MH with the presence of only one metabolic risk phenotype. We identified a mid (M) “gray zone” where a second group of GEMM SF individuals (N = 43) had a mean ALR of 4.0 ± 4.5 and presented between 2 to 7 metabolic risk phenotypes. For the third group (N = 40) with a mean (L)ALR of 0.6 ± 0.3 the participants presented between 2–8 metabolic risk factors. Table 3 and Figure 1 demonstrate these data by presenting the risk criteria cut-offs, the number of cardiometabolic risk phenotypes present in everyone and the percentage of these risk phenotypes within each of the three groups with AT dysfunction. Compared to the group with a (H)ALR, subjects from the (M) and (L)ALR groups had a much larger and more significant prevalence of IR and prediabetes.

When comparing the three different groups for the presence of AT dysfunction through a (H), (M) and (L)ALR, a clear picture emerges. There is a gradual and ascendant increase of the prevalence of their dysglycemic state, their IR state, increased triglycerides, their proinflammatory state and prediabetic state that can be easily correlated with their AT function. Through the biology and molecular actions of adiponectin and leptin, the ALR seems to offer a means to translate clinical biochemistry features into cellular steps for the transition between a healthy and unhealthy AT expansion in SF individuals. We could speculate that the (H)ALR group in our GEMM SF cohort represents a mild physiologic hypoxia during early healthy AT expansion, appropriate signaling for sufficient angiogenesis and small initial inflammatory cell infiltration. This induces an organized extracellular matrix (ECM) remodeling process for adequate expansion, involving functional adipocytes and the presence of predominantly M2 macrophages without fibrosis (Graphical Abstract).

In the presence of an excessive calorie-dense, palatable macronutrient intake there is adipocyte growth, which ultimately changes the healthy AT expansion through a progressive increase in ECM component synthesis, leading to a discrete and polarized macrophage phenotypic conversion, which characterizes the (M)ALR group. Chronic high caloric intake over time results in adipocyte hypertrophy, hypoxia and impaired ECM synthesis. The deleterious inflammatory response is fully triggered, predominantly by the presence of M1-polarized macrophages secreting a vast array of proinflammatory cytokines (mainly IL-6 and TNF-α). Due to severe hypoxia, a vicious cycle of persistent injury signaling and resultant impaired angiogenesis limits oxygen supply, subsequently triggering CLGSCI and massive proinflammatory macrophage infiltration, which characterizes the (L)ALR group (Graphical Abstract).

This scenario sets the landscape for excessive fibrosis, promoting significant AT hypertrophy, triggering the associated systemic immunometabolic dysfunction and leading to deleterious metabolic consequences such as T2DM, impaired angiogenesis, systemic inflammation, IR, endothelial dysfunction, altered lipid–lipoprotein metabolism and atherosclerosis [45,46] This same scenario could also be applicable to SF individuals with a (L)ALR independent of their AT accumulation and BMI measurements. It seems clear that AT dysfunction as a proxy for AT unhealthy expansion, measured through the ALR, is the common denominator that dictates early transition to immunometabolic impairments such as LGCSI, IR, postprandial proinflammatory response leading to dysglycemic states, prediabetes, lipid–lipoprotein abnormalities, and endothelial dysfunction phenotypes (Graphical Abstract). It appears that these phenotypes pertain to an approach that has not been exploited in full within the routine clinical setting [47,48]. These phenotypes are also considered CVD risk factors of immunometabolic origin [15], termed as nontraditional CVD risk factors (Table 1 and Table 2) [48,49]. It is also worth mentioning that this scenario may have individualized permissive factors and a strong genetic background to affect different individuals with different thresholds of fat accumulation. This has been implied in animal models where scientists have shown that there are potential differences in lipolysis by demonstrating that leptin-induced lipolysis may be regulated by nitric oxide (NO) [50].

As described in our results, we used heat maps to provide a descriptive representation of the postprandial comprehensive sequencing of miRNA in F/P plasma exosomal samples and a shotgun lipidomics dynamic process at 180′ to compare differences between female participants (N = 14) with (H) and (L)ALR (Table 4). We are only presenting the postprandial results 3 h after food ingestion, representing the peak of digestion. The lipidomic and miRNA heat maps show a fold increase of molecular lipid species and miRNA differential expression at time point 180′ for (L)ALR individuals as compared to (H)ALR ones (Figure 2 and Figure 4). Our preliminary results interestingly showed that the “hot brown” highest values in shotgun lipidomics and miRNA differential expression were found in participant 16 with a (L)ALR, and participant 21 with a (H)ALR. We performed volcano plots that enabled identification of lipid signatures with large fold changes, with the most biologically significant lipid species differentially expressed, also identifying miRNAs signatures with a trend for downregulation (Figure 3 and Figure 5). Figure 3 shows the specific classes of lipids differentially expressed between the (H) and (L)ALR at 180 min. These classes were PI [51], AC [52], Cer [53] and TAG [54]. The miRNA identified were hsa-miR-335-3p [51], hsa-miR-483-5p [52], hsa-miR-505-3p [53] and hsa-miR-584-5p [54] (Figure 5).

Female participants 16 and 21 seemed to have had the highest lipidomics and miRNA activities and expression profiles (Figure 2 and Figure 4). Participant 16 is a symptom-free metabolically unhealthy 35 year old female with AT dysfunction (ALR = 0.20), insulin resistance (Matsuda index of 2.7, HOMA-IR of 4.06), chronic subclinical inflammation (hsCRP of 51.8 mg/L) and HDL-C of 45 mg/dL, with a high risk of developing T2DM. Participant 21 is a symptom-free metabolically unhealthy 30 year old female with an ALR = 1.41, a Matsuda index of 2.6, fasting glucose levels of 101 mg/dL and 2 h-postprandial glucose levels of 155 mg/dL and fasting triglycerides of 166 mg/dL, which translates to a strong predisposition of developing a dysglycemic state [33]. Although our sample is too small for rigorous conclusions, these results illustrate examples of how we can characterize our future full dataset to screen plasma circulating molecular biomarkers and identify early signatures of risk for immunometabolic and cardiovascular disease and achieve a translational (from bench to bedside) and personalized medicine approach.

Finally, we obtained SKM and SQAT biopsies from six SF volunteers chosen from our previous fourteen SF adult females to perform whole-transcriptome analysis with total RNA sequencing (RNA-Seq). We obtained the F/P SKM (vastus lateralis) and SQAT biopsies from the thigh of each SF participant. We obtained our subcutaneous (SQ) tissue biopsy from the thigh instead of the abdominal SQ depot, and it has been documented that adipocyte formation is higher in the gluteofemoral compared to the abdominal SQ adipose depot when in vivo adipogenesis is directly measured [55,56]. In addition, thigh SQAT mass (but not abdominal SQAT mass) is weakly associated with a lower prevalence of metabolic syndrome and cardiometabolic risk [57]. Support for these findings is provided by studies showing that the relative release of palmitoleate, an insulin-sensitizing lipokine, is markedly higher from SQ gluteofemoral AT compared with SQ abdominal AT. Furthermore, by directly measuring adipogenesis, important information has been documented regarding preadipocyte and adipocyte formation being higher in the femoral compared to the abdominal SQ adipose depot [58,59,60]. Although this study comprises a small sample size (N = 6), the purpose was to obtain proof of concept data, which we performed by repeated measurements of key immunometabolic phenotypes that are related to body composition: adipose tissue biology, the insulin–glucose axis proinflammatory biomarkers and postprandial metabolism. This is a subset of only six female participants matched by gender and percentage of body fat, independent of BMI, weight or age but divided by (H) vs. (L)ALR; therefore, a weakness of the analysis is that perhaps a power analysis should have been done instead given the small sample size and the number of phenotypes compared between the (H) and (L)ALR groups. Although our results showed several not significant *p* values and some phenotypes, specifically BMI, showed no differences between the compared groups, we speculate that with a larger sample size, differences could have been detected.

The contrast of the linear combination of phenotypes between the mean (H) and (L)ALR groups showed that the ALR, IL-6, hsCRP and the AUC for insulin were significantly higher in the L(ALR) group, perhaps indicating the presence of a higher proinflammatory activity in the (L)ALR group (Table 6). We assigned participants with a (L)ALR (N = 2, mean 0.52, Table 5) < 1 as the at-risk group (RG) and participants with a (H)ALR (N = 4, mean 1.98, Table 5) > 1 as the control group (CG). The logarithmic fold change (FC) for RNA was calculated for 0′ and 180′ (F/P). We also contrasted the two groups (RG vs. CG) using the natural logarithmic FC to identify genes ranked by unadjusted *p*-values to obtain a probability measurement. Figure 6 shows that the three most relevant genes differentially expressed in SKM were *CCT6A* [61,62], *PMP22* [63] and *TSC22D1* [64]. Interestingly, Figure 7 shows that the hottest activity detected in the heatmap for SKM gene expression was *CCT6A* for adult pathways involving the immune system (B cells, CDK4, CDK8, monocytes and NK cells). Figure 8 shows that *TSC22D1* was the key centered gene associating a vast array of interactive pathways obtained through FunRich annotations for SKM transcript expression.

Figure 9 shows that the two most relevant genes differentially expressed in AT were *WNT5A* [65] and *FNDC1* [66,67]. Figure 10 demonstrates that the *MCM4* gene, involved with adult pathways involving the immune system (B cells, CDK4, CDK8, monocytes and NK cells), had the hottest activity detected in the heatmap for SQAT gene expression. Figure 11 shows that *MCM4* was the key centered gene associating a vast array of interactive pathways obtained through FunRich annotations for SQAT transcript expression [68]. Overall, Figure 6, Figure 7, Figure 8, Figure 9, Figure 10 and Figure 11 demonstrate a molecular workflow for bioinformatics analysis and provide a stepwise strategy to translate key transcriptomic/lipidomic expression into biological pathways and gene–gene expression interactions and associations of SKM and AT in the fasting and postprandial state.

## 5. Conclusions

Our study characterized F/P nonconventional biomarkers in blood and directly measured genomic and molecular pathways of F/P AT inflammation and other features of dysfunctional AT. We combined integrated, clinical and multiOMIC profiles from blood, AT and SKM, three tissues key to immunometabolic biology, and outlined novel methods and procedures in SF adults to identify biochemical pathways and potential regulatory networks. However, our data are cross-sectional, not longitudinal, therefore, it remains to be seen that the identification of early biomarkers are truly associated with risk factors with future abnormal metabolic symptoms. Nevertheless, by characterizing some of the earliest biomarkers of immunometabolic dysregulation, we can identify which subjects may have an unhealthy metabolism, therefore identifying risk factors that lead to the development of T2DM and CVD.

## Figures and Tables

**Table 1:** Demographic characteristics and clinical parameters for the adipose tissue dysfunction phenotype in our symptom-free GEMM volunteers.

Demographic Characteristics	Phenotypes and Cut-Offs (Mean ± SD)		
N = 124	Adipose Tissue (Dys)function		
Sympton-Free Participants (Metabolic Risk Criteria)	ALR > 1 (N = 84)	ALR < 1 (N = 40)	*p*
ALR	6.7 ± 10.0	0.6 ± 0.3	<0.0001
Age	37 ± 14	39 ± 14	0.3887
Weight (kg)	70 ± 15	84 ± 16	<0.0001
Waist Circunference (cm)	87 ± 12	99 ± 15	<0.0001
BMI (kg/m^2^)	27 ± 5	32 ± 6	<0.0001
% Fat Total	34 ± 9	40 ± 8	<0.0001
Systolic Pressure (mmHg) ≥ 130	111 ± 11	111 ± 14	0.7483
Diastolic Pressure (mmHg) ≥ 85	70 ± 10	72 ± 10	0.3103
Fasting Glucose (mg/dL) ≥ 100	97 ± 22	110 ± 29	0.0419
2-h Glucose (mg/dL) ≥ 140	127 ± 42	145 ± 54	0.0935
Triglycerides (mg/dL) ≥ 150	118 ± 51	150 ± 57	0.0007
HDL-cholesterol (mg/dL) < 40, Men; <50 Women	48 ± 14	42 ± 11	0.0313
High sensitive C-reactive protein (mg/L) (>90th percentile) *	10.0 ± 13.8	19.6 ± 22.2	0.0027
Whole body insulin resistance (Matsuda index) **	4.6 ± 4.0	2.7 ± 2.9	<0.0001

Absence of diagnosis or therapy of cardiometabolic diseases: T2D, hypertension, dyslipidemia, NAFLD, CKD, or CVD; or treatment with blood pressure, lipid, or diabetes medications.* hsCRP 90% percentile: >35.72 mg/L; ** Whole body insulin resistant: equal or lower than 2.5.

**Table 2:** Immunometabolic and postprandial parameters for the adipose tissue dysfunction phenotype in our symptom-free GEMM volunteers.

Immunometabolic and Postprandial Phenotypes	Phenotypes (Mean ± SD)		
N = 124	Adipose Tissue (Dys)function		
Sympton-Free Participants	ALR > 1 (N = 84)	ALR < 1 (N = 40)	*p*
Adiponectin (μg/mL)	32.7 ± 33.0	10.1 ± 5.2	<0.0001
Leptin (ng/mL)	8.7 ± 6.5	21.0 ± 18.4	<0.0001
TNFa (pg/mL)	5.0 ± 2.9	5.2 ± 2.8	0.6290
IL-6 (pg/mL)	9.1 ± 22.6	8.6 ± 9.5	0.0014
MCP-1 pg/mL	108.2 ± 143.9	107.1 ± 41.8	0.0544
PAI-1 (pg/mL)	34,169.0 ± 29,375.5	39,182.1 ± 30,956.3	0.2658
Fibrinogen (mg/dL)	107.7 ± 40.8	131.4 ± 73.4	0.0355
Fasting Insulin (microU/mL)	14.6 ± 18.6	25.2 ± 20.3	<0.0001
C-peptide (pg/mL)	1.2 ± 0.5	1.9 ± 0.9	<0.0001
Insulin 120′ [microU/mL]	64.8 ± 44.4	120.9 ± 74.4	<0.0001
HOMA-IR	3.6 ± 4.7	7.2 ± 6.5	<0.0001
Glucagon (pg)mL)	47.4 ± 64.2	46.5 ± 45.3	0.5168
GLP-1 (pg/mL)	13.0 ± 36.3	17.3 ± 44.3	0.8721
Leptin AUC (5h)	2598	6194	0.0002
Insulin AUC (5h)	16,847	28,158	0.0821
Glucose AUC (5h)	36,486	40,883	0.1124
Triglycerides AUC 5h	48,220	59,798	0.0025
GLP-1 AUC 5h	4743	5346	0.1306
Glucagon AUC 5h	18,487	17,435	0.1988
C-peptide AUC 5h	916.4	1246	0.0821

**Table 3:** Prevalence and percentages of parameters and cut-offs widely used to define metabolic risk and a healthy/unhealthy cardiometabolic profile (MH/MUH) in subjects with adipose tissue dysfunction determined by a mean (H) or (L)ALR.

	Prevalence and Percentage of Individuals with Risk Phenotypes		
	Group 1 (N = 41)	Group 2 (N = 43)	Group 3 (N = 40)
MH/MUH risk criteria and cut-offs	Mean ALR 9.5 ± 13.0	Mean ALR 4.0 ± 4.5	Mean ALR 0.6 ± 0.3
Diabetic A1c > 6.5	0 (0.0%)	3 (7.0%)	4 (10.0%)
Prediabetic A1c 5.7–6.4	1 (2.4%)	6 (14.0%)	7 (17.5%)
Matsuda Index < 2.5	4 (9.8%)	17 (39.5%)	25 (62.5%)
HOMA-IR > 2.6	12 (29.3)	16 (37.2%)	28 (70.0%)
hsCRP > 35.7	0 (0.0%)	4 (9.3%)	8 (20.0%)
Glucose > 100	0 (0.0%)	28 (65.1%)	16 (40.0%)
Triglycerides > 150	3 (7.3%)	17 (39.5%)	16 (40.0%)
HDL < 40 Men < 50 Women	16 (39.0%)	36 (83.7%)	33 (82.5%)
Dias BP > 85	1 (2.4%)	5 (11.6%)	4 (10.0%)
Sys BP > 130	0 (0.0%)	5 (11.6%)	4 (10.0%)
Waist > 88 Women - >102 Men	3 (7.3%)	26 (60.5%)	30 (75.0%)

**Table 4:** Demographics and clinical biochemistries in symptom-free adult volunteers (N = 14).

Demographic Characteristics	Mean Values for 14 Females			
	(H) ALR (N = 9)	SD (±)	(L) ALR (N = 5)	SD (±)
Adipo/Lep Ratio	2.2	1.1	0.5	0.4
Age (Yr)	38.5	11.8	32.6	13.0
% Fat Total	42.7	6.2	46.0	3.2
Weight (kg)	62.1	5.7	77.6	17.8
Waist Circumference (cm)	83.9	9.9	93.9	16.7
BMI (kg/m^2^)	26.8	3.1	32.8	7.8
Triglycerides (mg/dL)	119.8	42.2	145.0	60.1
HDL-Cholesterol (mg/dL)	41.4	9.6	47.8	9.4
Adiponectin (μg/mL)	24.9	15.5	7.0	2.8
Leptin (ng/mL)	11.1	3.9	21.0	11.3
Fasting Glucose (mg/dL)	87.0	6.9	86.2	8.4
Glucose 120′ (mg/dL)	121.2	21.8	107.0	7.0
Fasting Insulin (microU/mL)	7.1	4.2	17.3	9.5
Insulin 120′ (microU/mL)	59.5	42.4	101.7	42.2
Matsuda Index	6.6	4.1	2.9	1.3
HOMA-IR	1.5	0.9	3.7	2.0
PAI-1 (pg/mL)	42,867.5	31,186.8	56,408.4	57,940.0
IL-6 (pg/mL)	1.6	1.2	3.1	2.5
TNFa (pg/mL)	2.5	1.5	3.4	2.2
MCP-1 pg/mL	109.8	38.3	133.8	61.2

**Table 5:** Demographics and ALR measurements of each participant (N = 6).

Female ID (N = 6)	Gender	Age	% Fat	Adiponectin (μg/mL)	Leptin (ng/mL)	Adipo/Lep Ratio
MTY0003	F	49	34.5	18.37	7.41	2.48
MTY0007	F	38	36.2	12.13	5.42	2.24
MTY0006	F	33	50.3	23.73	11.50	2.06
MTY0014	F	45	49.9	16.96	15.03	1.13
Mean		41	42.7	17.80	9.84	1.98
SD (±)		7	8.5	4.77	4.29	0.59
MTY0009	F	20	43.2	8.46	8.93	0.95
MTY0010	F	35	47	3.12	35.27	0.09
Mean		28	45.1	5.79	22.10	0.52
SD (±)		10	2.7	3.78	18.62	0.61

**Table 6:** Contrast linear combination of variables between the CG and RG (N = 6).

	(H)ALR > 1			(L)ALR < 1					
Variable	Mean	SD (±)	SE	Mean	SD (±)	SE	Diff.	SE Diff.	*p*-Value
Waist Circumference (cm)	82.5	7.5	4.33	90	18.08	10.44	−7.5	11.3	0.543
BMI (kg/m^2^)	25.96	1.892	1.092	32.4	8.412	4.856	−6.43	4.978	0.265
% total fat	40.33	8.673	5.007	46.7	3.36	1.939	−6.36	5.37	0.301
Fat Mass kg	23.8	7.9	4.561	32.88	10.82	6.248	−9.08	7.736	0.305
Muscle Mass kg	34.28	1.718	0.991	37.18	11.05	6.384	−2.9	6.46	0.676
Triglycerides (mg/dL)	125.3	9.018	5.206	155.6	86.43	49.9	−30.3	50.17	0.578
Creatinin (mg/dL)	0.6	0.264	0.152	0.666	0.152	0.088	−0.06	0.176	0.724
Uric acid (mg/dL)	3.733	1.795	1.036	5.933	0.65	0.375	−2.2	1.102	0.116
BUN (mg/dL)	8	4	2.309	9.333	0.577	0.333	−1.33	2.333	0.598
Total Cholesterol (mg/dL)	162.3	80.22	46.31	156	11.13	6.429	6.333	46.76	0.898
HDL (mg/dL)	35	14.79	8.544	47.66	13.27	7.666	−12.6	11.47	0.331
LDL (mg/dL)	110	59.02	34.07	79	9.539	5.507	31	34.52	0.419
VLDL (mg/dL)	17.33	6.429	3.711	29.33	17	9.82	−12	10.49	0.316
Alt (U/L)	16.66	5.859	3.382	28.33	12.74	7.356	−11.6	8.096	0.223
Ast (U/L)	33.33	12.89	7.446	55.33	18.82	10.86	−22	13.17	0.17
Alk phos (U/L)	73.33	24.54	14.16	49.66	32.12	18.55	23.66	23.34	0.367
Adipo/lep Ratio	2.804	1.448	0.836	0.627	0.213	0.123	2.177	0.845	0.061 *
PAI-1 (pg/mL)	21,095	28,057	16,198	68,896	46,810	27,026	−4780	31,509	0.203
MCP-1 (pg/mL)	98.3	20.55	11.86	124.7	33.41	19.28	−26.4	22.64	0.307
IL-6 (pg/mL)	0.656	0.705	0.407	2.833	0.667	0.385	−2.17	0.56	0.017 **
TNF-a (pg/mL)	2.169	1.47	0.849	3.035	1.115	0.644	−0.86	1.065	0.461
hsCRP (mg/L)	0.058	0.029	0.017	0.304	0.165	0.095	−0.24	0.096	0.063 *
Matsuda Index	8.991	5.405	3.12	3.313	1.667	0.962	5.678	3.265	0.157
HOMA-IR	0.905	0.393	0.226	3.39	2.88	1.663	−2.48	1.678	0.212
AUC Glucose	350	61.55	35.53	310.9	2.155	1.244	39.08	35.56	0.333
AUC Insulin	134.8	61.86	35.71	282.8	87.22	50.36	−148	61.74	0.074 *
AUC GLP-1	304.8	52.32	30.21	470.3	236.1	136.3	−165	139.6	0.301

Statistically Significant *p*-Value: **; Marginally Significant *p*-value: *.

## Data Availability

The data presented in this study are available on request from the corresponding author.
